# Supplementary material for: Oribatid mites show that soil food web complexity and close aboveground-belowground linkages emerged in the early Paleozoic
Source: Commun Biol. 2019 Oct 22;2:387. doi: 10.1038/s42003-019-0628-7 (PMC6805910; doi:10.1038/s42003-019-0628-7)
Supplement: Supplementary file 1 — Supplementary Information [file 42003_2019_628_MOESM1_ESM.pdf]

Supplementary Figures

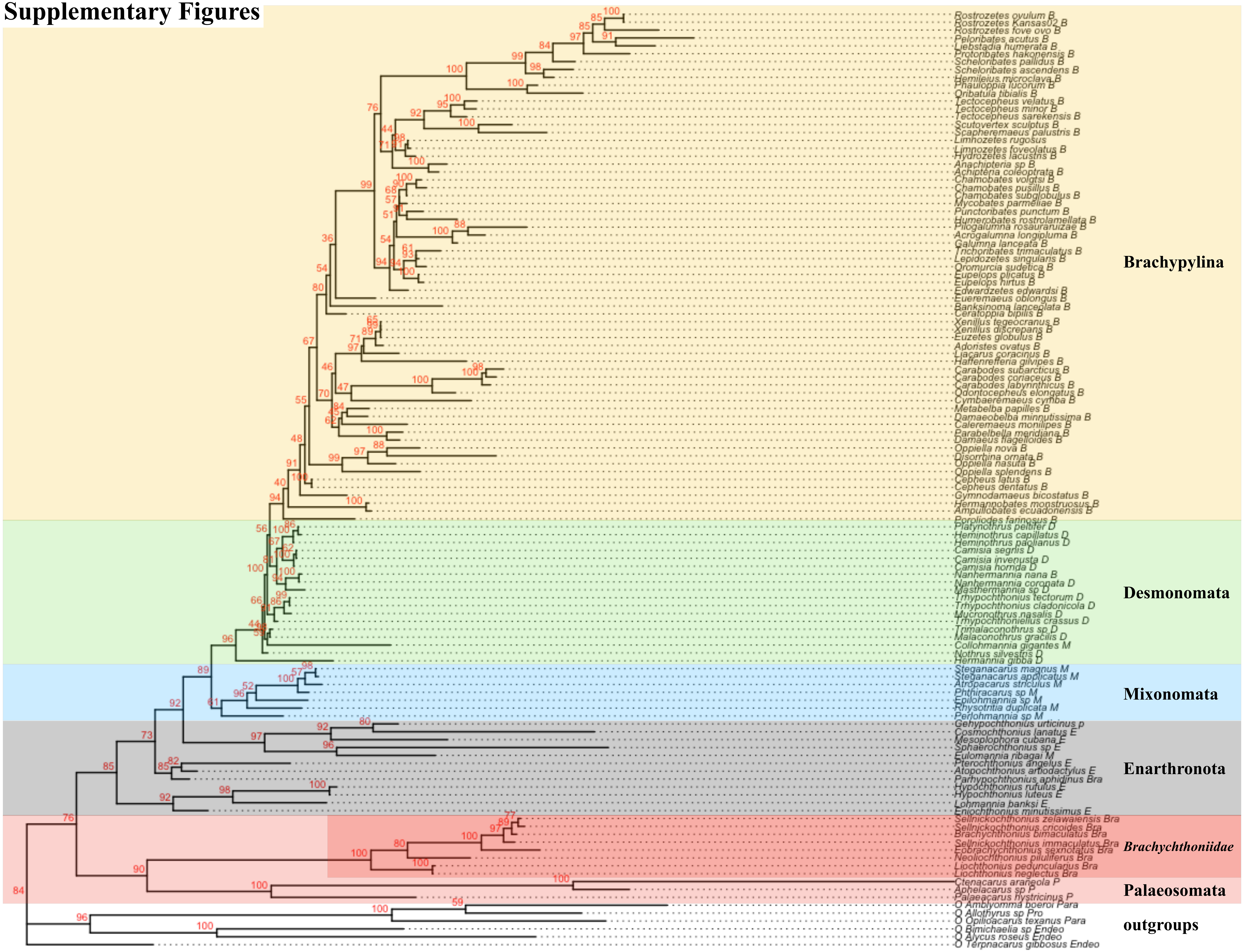

Supplementary Figure 1. Maximum Likelihood tree of the major groups of oribatid mites with 1000 bootstrap replicates.

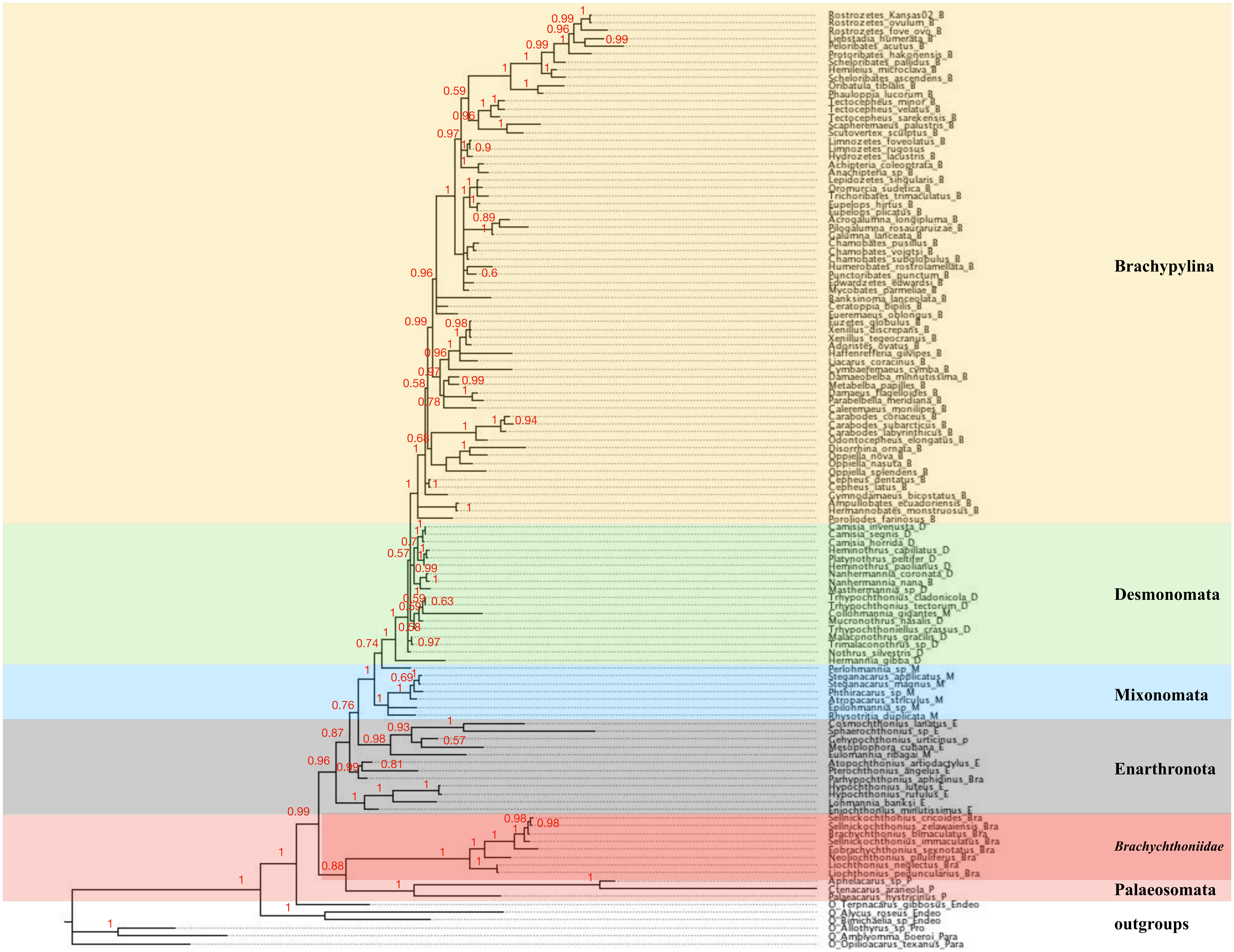

Supplementary Figure 2. Bayesien inference tree of the major groups of oribatid mites with posterior probabilities after 4 million generations.

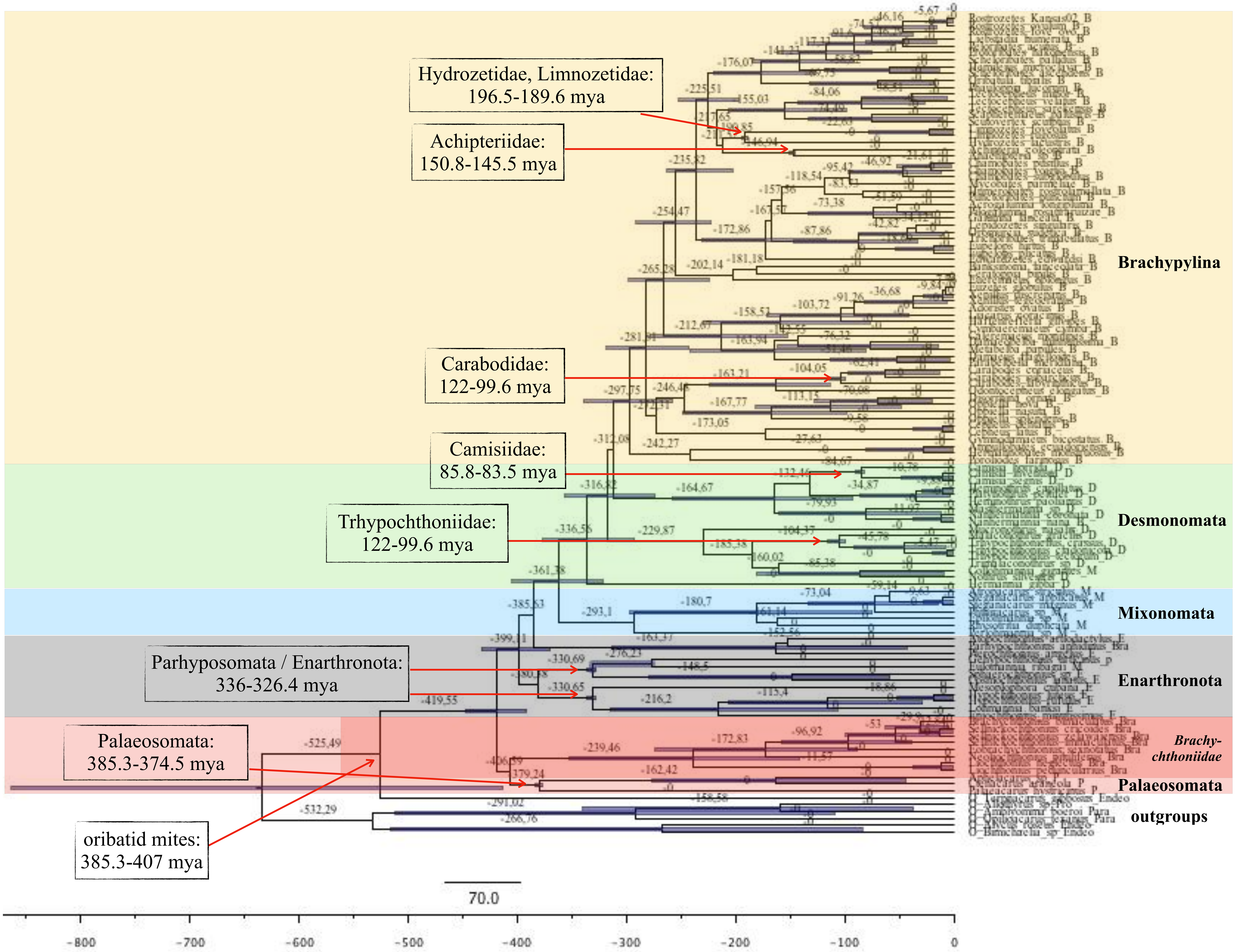

Supplementary Figure 3. BEAST phylogeny of the major groups of oribatid mites with estimated divergence times based on 9 fossil constraints. Calibration points are shown in boxes, for details refer to Supplementary Table 2.

Phylogeny of oribatid mites based on 18S rDNA. Phylogenetic trees were reconstructed with a 2,096 bp alignment of 18S rDNA of 113 oribatid mite species and three non-acariformes outgroup species (3 Parasitiformes). Maximum Likelihood (ML) analysis was performed in R using the pml function of the phangorn package, using the GTR+I+G model and 1,000 bootstrap replicates (Supplementary Figure 1). Bayesian Inference (BI) was conducted in MrBayes v3.2.6 using lset parameters nst=6 rates=invgamma and 4 million generations, all other parameters were set as default (Supplementary Figure 2).

The supercohorts Brachypylina and Desmonomata (=Nothrina) included 64 and 17 species, respectively, and were recovered with 94 and 96 percent bootstrap support, respectively (posterior probabiliy=1 for each). The tropical genus *Rostrozetes* represents Haplozetidae, because no sequences of temperate species were available, but body sizes and stable isotope data of three European species. The monotypic superfamily Collohmanniidae clustered within *Trimalaconothridae.* A similar relationship of *Collohmannia gigantea* was also recovered in Pachl et al. (2017)[1], also with equivocal node support.

The supercohort Mixonomata was recovered with 92 percent bootstrap (bs) support (posterior probability (pp)=1) and included three Phthiracaroidea, one Euphthiracaroidea and also included the superfamily Epilohmannioidea which agrees with other studies[1-3]. The position of Perlohmannioidea differed in the ML and BI, being basal to Mixonomata in ML and ancestral to Desmonomata in the BI tree, the later is consistent with the BI analysis of a concatenated 18S+28S rDNA dataset[1] and the BEAST analysis in this study (Supplementary Figure 3).

The supercohort Endeostigmata was paraphyletic in both analyses (ML and BI), resolving into three monophyletic clades. First, the superfamily Hypochthonioidea (bs=92, pp=1) including two species of the genus *Hypochthonius*, *Eniochthonius* and *Lohmannia* which is in agreement with Krantz and Proctor (2009)[4] and with Dabert et al. (2010)[5] but not with Pachl et al. (2017) which group Eniochthoniidae basal to Mesoplophoridae. Second, the superfamily Protoplophoridae (bs=97, pp=1), has not been recovered in this and previous molecular phylogenies, mainly due to strong differences in taxon sampling, e.g. of 14 enarthronote taxa in Pachl et al. (2017) of which only six overlap with this study. However, according to Krantz and Proctor (2009) Cosmochthoniidae and Sphaerochthoniidae are part of the superfamily Protoplophoroidea but, in contrast, here Mesoplophoridae are not part of Hypochthoniidae. The position of Eulohmanniidae is traditionally close to Mixonomata but has been recovered as sister taxon to *Gehypochthonius* in Pachl et al. (2017). Consistent with Schaefer et al. (2010) and Pachl et al. (2017), the supercohort Parhyposomata, here represented by the genera *Gehypochthonius* and *Parhypochthonius* were not recovered but clustered among other enarthronote mites. Third, the superfamily Atopochtonioidea was recovered (bs=85, pp=0.99) and included the genera *Atopochthonius* and *Pterochthonius* and additionally the parhyposomatid genus *Parhypochthonius* (Parhyposomata).

Notably, the enarthronote superfamily Brachychthonioidea (bs=100, pp=1) clustered with medium support (bs=90, pp=0.88) as sister group to Palaeosomata (bs=96, pp=1) which has not been shown before. Klimov & OConnor (2013)[6] included one Palaeosomata and two Brachychthoniidae in their study and recovered them as sister taxa as well, but not as monophyletic. This indicates that these taxa require more attention to resolve the early-derived relationships within oribatid mites and among acariformes.

<u>The choice of outgroups and their effects on molecular clock estimates:</u> The topology is robust and the mean molecular divergence time estimate of ~415 mya (ranging from 371-447 mya) between [Palaeosomata + Brachychthoniidae] and [Enarthronota + remaining oribatid mites] is reasonable and predating the oldest oribatid mite fossils (i.e., Enarthronota) only by about 25 my. The phylogeny among basal Acariformes and of Acari among Chelicerata is still not resolved with the molecular data currently available, irrespective of the number of genes and the methods used (e.g. secondary structures of rDNA to improve alignment quality) to improve the phylogeny on the base of Acariformes/Acari (e.g. Pepato & Klimov 2015). This study concentrated on nodes within the group of oribatid mites, and used only Acari as outgroups to infer the basal relationships and the origin of the stem group of oribatid mites. The position of Acariformes among Chelicerates is out of the scope of this study and adding more outgroups would not change the internal topology and age of internal nodes of the investigated ingroup (i.e. oribatid mites without Astigmata).

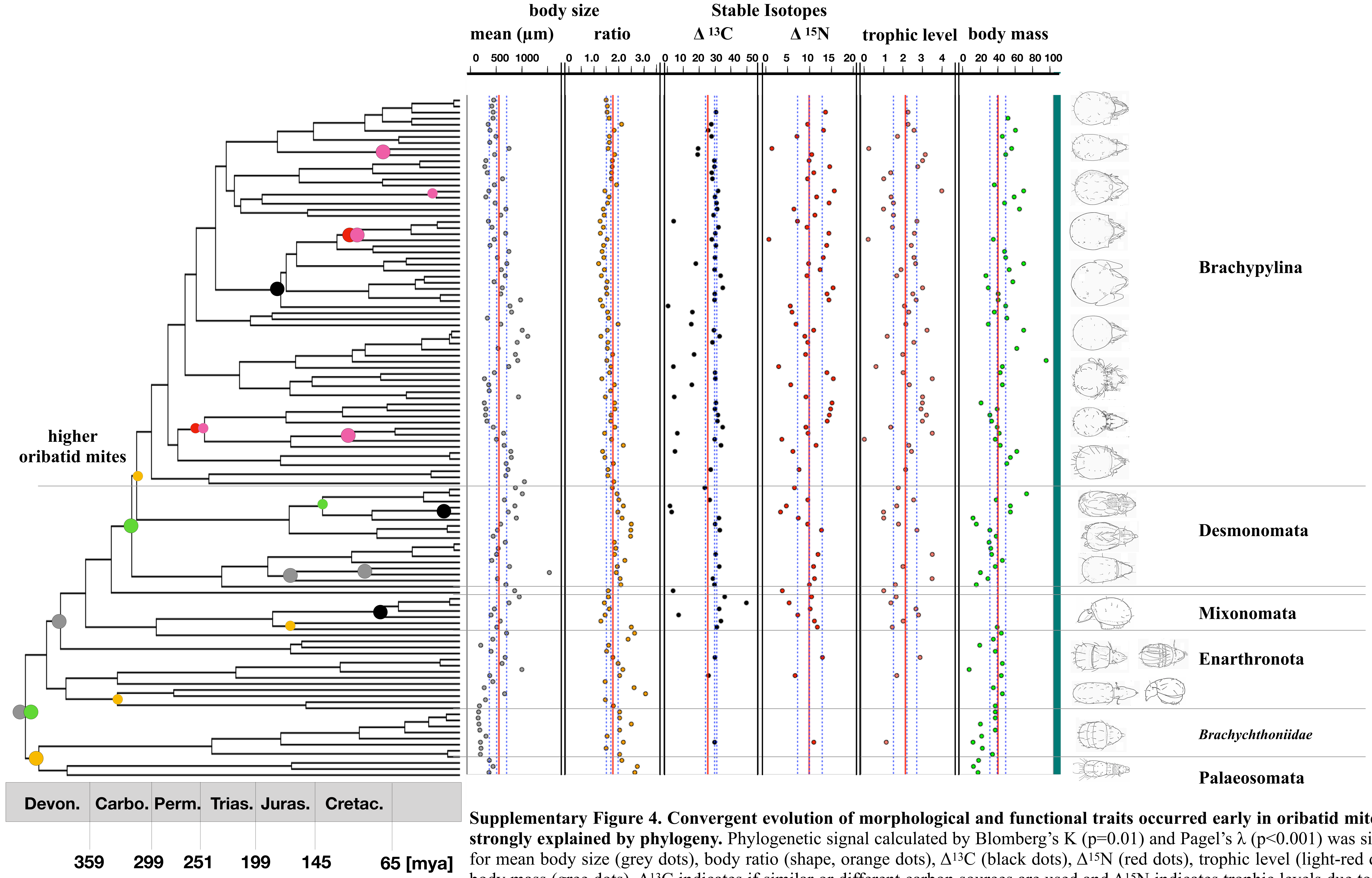

Supplementary Figure 4. Convergent evolution of morphological and functional traits occurred early in oribatid mites and is strongly explained by phylogeny. Phylogenetic signal calculated by Blomberg's K (p=0.01) and Pagel's λ (p<0.001) was significant for mean body size (grey dots), body ratio (shape, orange dots), $\Delta^{13}C$ (black dots), $\Delta^{15}N$ (red dots), trophic level (light-red dots) and body mass (gree dots). $\Delta^{13}C$ indicates if similar or different carbon sources are used and $\Delta^{15}N$ indicates trophic levels due to nitrogen enrichment in higher levels.

Circles on nodes highlight nodes which traits significantly differ on decending clades (PIC), size of points indicate if the trait increased (large) or decreased (small). Trait distribution is mapped along the phylogeny next to species. Red lines in trait columns show the mean distribution, dashed blue lines show the 25% and 75% quantiles and median of the respective trait. Images of common body forms within each taxonomic group are provided on the right). Body mass was calculated after Caruso & Migliorini (2009)[7] using body length and width of drawings in Weigmann 2006[8].

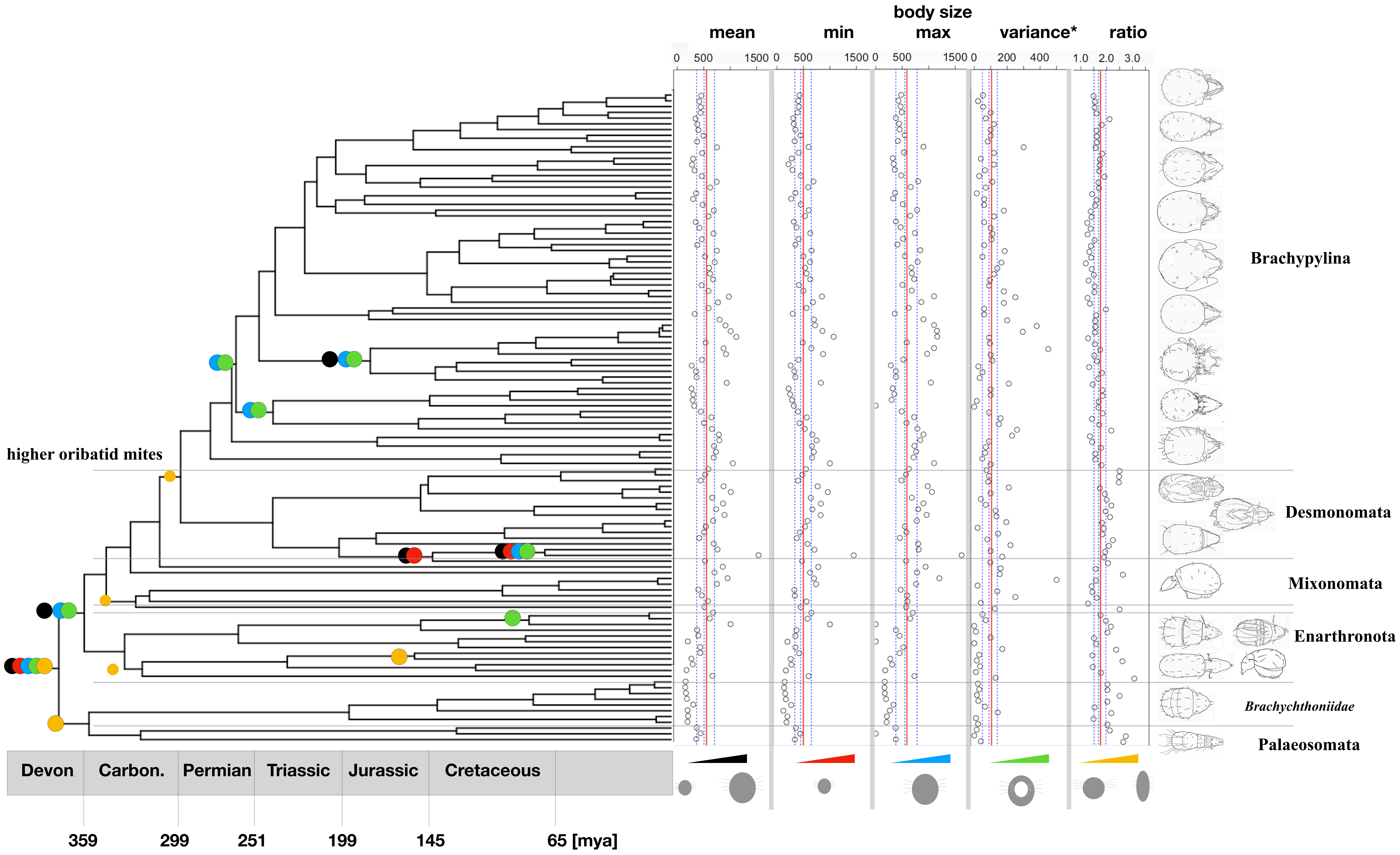

Supplementary Figure 5. Convergent evolution of body size related traits occurred early in oribatid mites and is strongly explained by phylogeny. Phylogenetic signal calculated by Blomberg's K (p=0.01) and Pagel's λ (p<0.001) was significant for mean (K=0.34, λ=0.78), minimum (min: K=0.35, λ=0.79), maximum size (max: K=0.0.37, λ=0.73), variance (λ=0.25, p<0.05) and ratio (body length/width: K=0.7, λ=0.93). Circles on nodes indicate significant trait divergence calculated by phylogenetic independent contrast (PIC) (black=mean size, red=minimum size, blue=maximum size, green= variance, orange=ratio). Trait distribution is mapped along the phylogeny next to species. Red lines in trait columns show the mean distribution, dashed blue lines show the 25% and 75% quantiles and median of the respective trait. Images of common body forms within each taxonomic group are provided on the right).; * indicates significance only for Pagel's λ.

Supplementary Tables

Supplementary Table 1. Taxonomic overview of species included in the phylogeny and NCBI accession numbers. All six, commonly recognized, groups (infraorders) of or oribtatid mites were covered[9,10], and three acariform (Endeostigmata) and three parasitiform mites were included as outgroups for the phylogenetic tree. The majority of oribatid mite species belong to the infraorder Brachypylina (=higher oribatid mites) and represents a balanced taxon sampling across all infraorders, because of 10,000 described oribatid mites species, about ~8,000 belong to Brachypylina[11]. When available on NCBI, more than ones species per genus were included when available on NCBI (highlighted in grey). The dataset included 103 oribatid mite species of Brachypylina (n=63), Desmonomata (n=17), Mixonomata (n=9), Enarthronota (n=17, including 8 Brachychthoniidae), Parhyposomata (n=2) and Palaeosomata (n=3).

infraorder	superfamily	family	genus	species (available as 18S sequence)	Accno (NCBI)
Brachypylina	Achipteroidea	Achipteriidae	Achipteria	*Achipteria_coleoptrata*	EF091418
		Achipteriidae	Anachipteria	*Anachipteria_sp*	JQ000048
			Lepidozetes	*Lepidozetes_singularis*	EU432193
	Ameroidea	Caleremaeidae	Caleremaeus	*Caleremaeus_monilipes*	MK630361
		Carabodidae	Carabodes	*Carabodes_coriaceus*	EF093787
				Carabodes_labyrinthicus	KX397629
				Carabodes_subarcticus	EF091429
			Odontocepheus	*Odontocepheus_elongatus*	EU432200
		Tectocepheidae	Tectocepheus	*Tectocepheus_minor*	EF093778
				Tectocepheus_sarekensis	EF093777
				Tectocepheus_velatus	EF093780
	Cepheoidea	Cepheidae	Cepheus	*Cepheus_dentatus*	MK630354
				Cepheus_latus	EU432206
	Ceratozetoidea	Ceratozetidae	Edwardzetes	*Edwardzetes_edwardsi*	MH198178
			Oromurcia	*Oromurcia_sudetica*	EU432194
			Trichoribates	*Trichoribates_trimaculatus*	EU432195
		Chamobatidae	Chamobates	*Chamobates_pusillus*	EU432188
				Chamobates_subglobulus	EU432190
				Chamobates_voigtsi	EU432189
		Euzetidae	Euzetes	*Euzetes_globulus*	AF022030
		Humerobatidae	Humerobates	*Humerobates_rostrolamellata*	EU432196

Supplementary Table 1 continued

	Mycobatidae	Mycobates	*Mycobates_parmeliae*	EU432191
	Punctoribatidae	Punctoribates	*Punctoribates_punctum*	XX123456
Cymbaeremaeoidea	Cymbaeremaeidae	Cymbaeremaeus	*Cymbaeremaeus_cymba*	EU432201
		Scapheremaeus	*Scapheremaeus_palustris*	EU433989
Damaeoidea	Belbodamaeidae	Damaeobelba	*Damaeobelba_minutissima*	MH198179
	Damaeidae	Damaeus	*Damaeus_flagelloides*	KR081608
		Metabelba	*Metabelba_papilles*	MH198180
		Parabelbella	*Parabelbella_meridiana*	KR081627
Eremaeoidea	Eremaeidae	Eueremaeus	*Eueremaeus_oblongus*	GQ864287
Galumnoidea	Galumnidae	Acrogalumna	*Acrogalumna_longipluma*	GQ864304
		Galumna	*Galumna_lanceata*	EU432197
		Pilogalumna	*Pilogalumna_rosauraruizae*	KJ423065
Gustavioidea	Liacaridae	Adoristes	*Adoristes_ovatus*	GQ864286
		Liacarus	*Liacarus_coracinus*	KR081619
	Tenuialidae	Haffenrefferia	*Hafenre fferia_gilvipes*	MK630363
Gustavioidea	Xenillidae	Xenillus	*Xenillus_discrepans*	EU432203
			Xenillus_tegeocranus	KR081637
	Ceratoppiidae	Ceratoppia	*Ceratoppia_bipilis*	EU432204
Gymnodamaeoidea	Gymnodaemaeidae	Gymnodamaeus	*Gymnodamaeus_bicostatus*	GQ864285
Hermannielloidea	Hermanniellidae	Ambullopates	*Ampullobates_ecuadoriensis*	KR081601
		Hermannobates	*Hermannobates_monstruosus*	KR081617
Hydrozetoidea	Hydrozetidae	Hydrozetes	*Hydrozetes_lacustris*	EU433987
	Limnozetidae	Limnozetes	*Limnozetes_foveolatus*	KX397634
			Limnozetes_rugosus	KX397636
Licneremaeoidea	Scutoverticidae	Scutovertex	*Scutovertex_sculptus*	GQ864305
Liodoidea	Liodidae	Poroliodes	*Poroliodes_farinosus*	EF203779
Oppioidea	Oppiidae	Disorrhina	*Disorrhina_ornata*	MH198181
		Oppiella	*Oppiella_nasuta*	MK630355
			Oppiella_nova	KR081626
			Oppiella_splendens	MK630356
	Thyrisomidae	Banksimona	*Banksinoma_lanceolata*	MK630359

Supplementary Table 1 continued

	Oripodoidea	Haplozetidae	Peloribates	*Peloribates_acutus*	AB818529
			Rostrozetes	*Rostrozetes_fove_ovo*	MK630358
				Rostrozetes_Kansas02	MK630357
				Rostrozetes_ovulum	HM070342
		Hemileiidae	Hemileus	*Hemileius_microclava*	KR081616
		Liebstadiidae	Liebstadia	*Liebstadia_humerata*	KR081620
		Oribatulidae	Oribatula	*Oribatula_tibialis*	EU433990
			Phauloppia	*Phauloppia_lucorum*	EU432198
		Protoribatidae	Protoribates	*Protoribates_hakonensis*	AB818528
		Scheloribatidae	Scheloribates	*Scheloribates_ascendens*	EU432199
	Phenopelopoidea	Phenopelopidae	Eupelops	*Eupelops_hirtus*	EF093782
				Eupelops_plicatus	EF093782
Desmonomata	Crotonioidea	Camisiidae	Camisia	*Camisia_horrida*	EU432207
				Camisia_invenusta	EU432208
				Camisia_segnis	EU432209
			Heminothrus	*Heminothrus_capillatus*	GQ864288
				Heminothrus_paolianus	EF091423
			Platynothrus	*Platynothrus_peltifer*	EF091422
		Nothridae	Nothrus	*Nothrus_silvestris*	EF091425
	Hermannioidea	Hermanniidae	Hermannia	*Hermannia_gibba*	EF091426
	Nanhermannioidea	Nanhermanniidae	Masthermannia	*Masthermannia_sp*	KY922217
			Nanhermania	*Nanhermannia_coronata*	EF091421
				Nanhermannia_nana	KR081624
	Trhypochthonioidea	Malaconothridae	Malaconothrus	*Malaconothrus_gracilis*	EF091424
			Trimalaconothrus	*Trimalaconothrus_sp*	EU43221
		Trhypochthoniidae	Mucronothrus	*Mucronothrus_nasalis*	EF081299
			Trhypochthoniellus	*Trhypochthoniellus_crassus*	EF081300
		Trhypochthoniidae	Trhypochthonius	*Trhypochthonius_cladonicola*	JQ000047
				Trhypochthonius_tectorum	AF022041
Mixonomata	Collohmannioidea	Collohmanniidae	Cohllohmannia	*Collohmannia_gigantes*	KR081604
	Epilohmannioidea	Epilohmanniidae	Epilohmannia	*Epilohmannia_sp*	EU432213
	Eulohmannioidea	Eulohmanniidae	Eulohmannia	*Eulomannia_ribagai*	EU432211
	Euphtiracaroidea	Eupthiracaridae	Rhysotritia	*Rhysotritia_duplicata*	EF091417
	Perlohmannioidea	Perlohmanniidae	Perlohmannia	*Perlohmannia_sp*	EU432212
	Phthiracaroidea	Phthiracaridae	Phthiracarus	*Phthiracarus_sp*	KR081629
		Steganacaridae	Atropacarus	*Atropacarus_striculus*	EF091416
			Steganacarus	*Steganacarus_applicatus*	GQ864301
				Steganacarus_magnus	AF022040

Supplementary Table 1 continued

Enarthronota	Atopochthonoidea	Atopochthoniidae	Atopochthonius	*Atopochthonius_artiodactylus*	EU432216
		Pterochthoniidae	Pterochthonius	*Pterochthonius_angelus*	EU432214
	Brachychthonioidea	Brachychthoniidae	Brachychthonius	*Brachychthonius_bimaculatus*	MK630360
			Eobrachychthonius	*Eobrachychthonius_sexnotatus*	MK630362
			Liochthonius	*Liochthonius_neglectus*	MK630364
				Liochthonius_peduncularius	MK630365
			Neoliochthonius	*Neoliochthonius_piluliferus*	MK630366
			Sellnickochthonius	*Sellnickochthonius_cricoides*	MK630367
				Sellnickochthonius_immaculatus	MN065455
				Sellnickochthonius_zelawaiensis	MH198174
	Cosmochthonioidea	Cosmochthoniidae	Cosmochthonius	*Cosmochthonius_lanatus*	JN585919
		Sphaerochthoniidae	Sphaerochthonius	*Sphaerochthonius_sp*	JN585916
	Hypochthonoidea	Eniochtoniidae	Eniochthonius	*Eniochthonius_minutissimus*	EF091428
		Hypochthoniidae	Hypochthonius	*Hypochthonius_luteus*	EU152475
				Hypochthonius_rufulus	EF091427
	Lohmannioidea	Lohmanniidae	Lohmannia	*Lohmannia_banksi_E*	AF022036
	Mesoplophoroidea	Mesoplophoridae	Mesoplophora	*Mesoplophora_cubana*	EU432217
Parhyposomata	Parhypochthonioidea	Gehypochthoniidae	Gehypochthonius	*Gehypochthonius_urticinus*	EU433994
		Parhypochthoniidae	Parhypochtonius	*Parhypochthonius_aphidinus*	EU432215
Palaeosomata	Ctenacaroidea	Aphelacaridae	Aphelacarus	*Aphelacarus_sp*	DQ648879
		Ctenacaridae	Ctenacarus	*Ctenacarus_araneola*	EU433991
	Palaeacaroidea	Palaeacaridae	Palaeacarus	*Palaeacarus_hystricinus*	EF204472
Acariformes	Endeostigmata	Alycidae	Alycus	*Alycus_roseus*	GQ864294
outgroups			Bimicaelia	*Bimichaelia_sp*	GQ864295
		Terpnacaridae	Terpnacarus	*Terpnacarus_gibbosus*	AY620904
Parasitiformes	Holothyrida	Holothyroidea	Allothyrus	*Allothyrus_sp*	AF018655
outgroups	Ixodidae	Amblyomminae	Amblyomma	*Amblyomma_boeroi*	FJ464420
	Opilioacariformes	Opilioacarida	Opilioacarus	*Opilioacarus_texanus*	AF115375

Supplementary Table 2. Taxonomic assignment, geological age of fossil oribatid mites, and prior settings used in the molecular clock analysis.

	family	species (available as 18S sequence)	fossil specimen	geological epoch	time (mya)	reference	exponential calibration prior in BEAST
**Brachypylina**	Achipteriidae	*Achipteria_coleoptrata*<br>*Anachipteria_sp*	*Achipteria (?) obscura*	Tithonian	150.8-145.5	Krivolutsky & Krassilov 1977[12]	mean:2.0<br>offset:145.0
	Carabodidae	*Carabodes_coriaceus*<br>*Carabodes_labyrinthicus*<br>*Carabodes_subarcticus*	*Cretaceobodes martinezae*	Albian	122-99.6	Arillo et al. 2008[13]	mean:7.0<br>offset:99.0
	Hydrozetidae<br>Limnozetidae	*Hydrozetes_lacustris*<br>*Limnozetes_foveolatus*<br>*Limnozetes_rugosus*	*Hydrozetes sp.*	Sinemurian	196.5-189.6	Sivhead & Wallwork 1978[14]	mean:2.0<br>offset:189.0
**Desmonomata**	Camisiidae	*Camisia_horrida*<br>*Camisia_invenusta*<br>*Camisia_segnis*	*Eocamisia sukatshevae*	Santonian	85.8-83.5	Bulanova-Zachvatkina 1974[15]	mean:3.0<br>offset:82.0
	Trhypochthoniidae	*Mucronothrus_nasalis*<br>*Trhypochthoniellus_crassus*<br>*Trhypochthonius_cladonicola*<br>*Trhypochthonius_tectorum*	*Trhypochthonius lopezvallei*	Albian	122-99.6	Arillo et al. 2012[16]	mean:7.0<br>offset:99.0
**Parhyposomata / Enarthronota**	Cosmochthoniidae<br>Gehypochthoniidae<br>Sphaerochthoniidae<br>Eulohmanniidae	*Cosmochthonius_lanatus*<br>*Gehypochthonius_urticinus*<br>*Sphaerochthonius_sp*<br>*Eulomannia_ribagai*	*Carbochthonius antrimensis*<br>*Gehypochthonimimus hibernicus*	Brigantian	336-326.4	Subías & Arillo 2002[17]	mean:3.0<br>offset:328.0

Supplementary Table 2 continued

**Enarthro-nota**	Hypochthoniidae	*Hypochthonius_luteus*<br>*Hypochthonius_rufulus*	*Paleohypochthonius jerami*	Brigantian	336-326.4	Subías & Arillo 2002[17]	mean:3.0<br>offset:328.0
	Mesoplophoridae	*Mesoplophora_cubana*	*Archaeoplophora bella*				
	Eniochtoniidae	*Eniochthonius_minutissimus*					
	Lohmanniidae	*Lohmannia_banksi*					
**Palaeoso-mata**	Aphelacaridae	*Aphelacarus_sp*	*Monoaphelacarus carboniferus*	lower Frasnian	385.3-374.5	Subías & Arillo 2002[17], Norton et al. 1988[18]	mean:3.0<br>offset:377.0
	Ctenacaridae	*Ctenacarus_araneola*	*Ctenacaronychus nortoni*				
	Palaeacaridae	*Palaeacarus_hystricinus*	*Paleoctenacarus simmsoi*				
**oribatid mites**				Emsian<br>Givetian	407-397.5<br>385.3-391.8	Shear et al. 1984[19]	mean:5.0<br>offset:384.0

Supplementary Table 3. Fossil data that decreased the likelihoods and posterior probabilites and were not used in the final molecular clock analyses. Fossil dates either strongly underestimated the age of species or could not be applied because to few specimens of the genus or family were present in the dataset.

	family	species (available as 18S sequence)	fossil specimen	geological epoch	time (mya)	reference
Brachypylina	Cepheoidea	*Cepheus dentatus*	*Eupterotegaeus bitranslamellatus*	Albian	122-99.6	Arillo et al. 2012[16]
		Cepheus latus	*Ommatocepheus nortoni*			
	Cymbae-remaeidae	*Cymbaeremaeus cymba*	*Ametroproctus valeiae*	Albian	122-99.6	Arillo et al. 2012[16]
		Scapheremaeus palustris	*Scapheremaeus*	late Eocene	42-37	O'Dowd et al. 1991[20]
	Eremaeidae	*Eueremaeus oblongus*	*Eremaeus denaius*	Oligocene/ Miocene	25	Woolley 1971[21]
	Galumnidae	*Acrogalumna longipluma*<br>*Galumna lanceata*<br>*Pilogalumna rosauraruizae*		middle Eocene	42-49	O'Dowd et al. 1991[20]
	Oppiidae	*Disorrhina ornata*	*Dissorhina paleokrasica* n. sp., *D. nuda* n. sp.	late Pliocene	3.8, 2.7	Mico 2015[22]
		Oppiella nasuta<br>*Oppiella nova*<br>*Oppiella splendens*	*Oppia hurdi*	Oligocene/ Miocene	25	Woolley 1971[21]
	Scutoverticidae	*Scutovertex sculptus*	*Hypovertex hispanicus (Scutoverticidae)*	Albian	122-99.6	Arillo et al. 2012[16]
		Phauloppia lucorum	*Phauloppia*	middle Eocene	42-49	O'Dowd et al. 1991[20]
Des.	Hermanniidae	*Hermannia gibba*	*Hermannia sellnicki* n. sp.	Lutetian	~44	Norton 2006[23]
Mixonomata	Collohmanniidae	*Collohmannia gigantes*	*Collohmannia schusteri n. sp.*	Lutetian	~44	Norton 2006[23]
	Phthiracaroidea	*Phthiracarus sp*<br>*Atropacarus striculus*<br>*Steganacarus applicatus*<br>*Steganacarus magnus*		Carboniferous trace fossils (coprolites)	359.2-299	Labandeira et al. 1997[24]
	Euphtiracarid.	*Rhysotritia duplicata*				

Supplementary Table 4. Summary of morphological traits (body size and body shape) used in this study. Minimum and maximum size values were collected from Weigman (2006)[8] if not stated otherwise in the reference column. Body shape (ratio of length/width) was obtained by measuring drawings of species from literature. If sequences were not assigned to species (i.e., sp.) or the exact species was not available as drawing, measurements were taken from a congeneric species as listed in the column references. Sequences of the species of the genus *Haplozetes* were not available at GenBank and were replaced by species of the genus *Rostrozetes* .

		body size [µm]				body form	
**infraorder**	**species (available 18S sequence)**	**mean**	**minimum**	**maximum**	**variance**	**(ratio= length/width)**	**reference**
Brachypylina (higher oribatid mites)	*Achipteria_coleoptrata*	590	530	650	120	1.41	
	Anachipteria_sp	690	600	780	180	1.38	*Anachipteria nitens*
	Lepidozetes_singularis	460	415	505	90	1.53	
	Caleremaeus_monilipes	355	330	380	50	1.82	
	Carabodes_coriaceus	645	565	725	160	1.43	
	Carabodes_labyrinthicus	505	430	580	150	1.71	
	Carabodes_subarcticus	445	400	490	90	1.84	
	Odontocepheus_elongatus	655	525	785	260	2.18	
	Tectocepheus_minor	280	220	340	120	1.73	Dogan & Ayyildiz 2000[25]
	Tectocepheus_sarekensis	327.5	295	360	65	1.71	Knülle 1953[26]
	Tectocepheus_velatus	300	280	320	40	1.74	Dogan & Ayyildiz 2000[25]
	Cepheus_dentatus	795	750	840	90	1.44	
	Cepheus_latus	785	670	900	230	1.35	
	Edwardzetes_edwardsi	770	680	860	180	1.35	
	Oromurcia_sudetica	677.5	630	725	95	1.3	
	Trichoribates_trimaculatus	620	560	680	120	1.5	*Trichoribates novus*
	Chamobates_pusillus	420	370	470	100	1.38	
	Chamobates_subglobulus	685	630	740	110	1.26	
	Chamobates_voigtsi	350	320	380	60	1.25	
	Euzetes_globulus	1115	1070	1160	90	1.28	
	Humerobates_rostrolamellata	747.5	655	840	185	1.33	
	Mycobates_parmeliae	462.5	410	515	105	1.53	
	Punctoribates_punctum	380	350	410	60	1.39	
	Cymbaeremaeus_cymba	745	690	800	110	1.68	
	Scapheremaeus_palustris	465	450	480	30	1.91	
	Damaeobelba_minnutissima	272.5	260	285	25	1.32	
	Damaeus_flagelloides	935	830	1040	210	1.46	

Supplementary Table 4 continued

Metabelba_papilles	465	410	520	110	1.63	
Parabelbella_meridiana	364.5	348	381	33	1.67	
Eueremaeus_oblongus	590	560	620	60	1.97	*Eueremaeus silvestris*
Acrogalumna_longipluma	707.5	625	790	165	1.19	
Galumna_lanceata	600	530	670	140	1.42	
Pilogalumna_rosauraruizae	525	500	550	50	1.4	*Pilogalumna crassiclava*
Adoristes_ovatus	537.5	490	585	95	1.54	
Liacarus_coracinus	875	650	1100	450	1.75	
Haffenrefferia_gilvipes	920	870	970	100	1.52	
Xenillus_discrepans	1007.5	860	1155	295	1.54	
Xenillus_tegeocranus	910	720	1100	380	1.56	
Ceratoppia_bipilis	800	700	900	200	1.55	
Gymnodamaeus_bicostatus	695	660	730	70	1.78	
Ampullobates_ecuadoriensis	688.5	664	713	49	1.56	
Hermannobates_monstruosus	730	697	763	66	1.56	
Hydrozetes_lacustris	480	450	510	60	1.55	
Limnozetes_foveolatus	300	270	330	60	1.61	*Limnozetes ciliatus*
Limnozetes_rugosus	357.5	350	365	15	1.44	
Scutovertex_sculptus	625	590	660	70	1.7	
Poroliodes_farinosus	1050	1000	1100	100	1.8	
Disorrhina_ornata	300	250	350	100	1.84	
Oppiella_nasuta	292.5	285	300	15	1.69	
Oppiella_nova	270	220	320	100	1.83	
Oppiella_splendens	320	320			1.68	*Oppiella escotata*
Banksinoma_lanceolata	330	300	360	60	1.59	
Peloribates_acutus	440	390	490	100	1.61	*Peloribates europaeus*
Rostrozetes_fove_ovo	442	415	469	54	1.53	*Haplozetes vindobonensis*
Rostrozetes_Kansas02	416.5	405	428	23	1.54	*Haplozetes elegans*
Rostrozetes_ovulum	452.5	426	479	53	1.49	*Haplozetes tenuifusus*
Hemileius_microclava	376	335	417	82	1.62	
Liebstadia_humerata	345	310	380	70	2.11	
Oribatula_tibialis	470	410	530	120	1.83	
Phauloppia_lucorum	750	600	900	300	1.57	

Supplementary Table 4 continued

Brachy-pylina	*Protoribates_hakonensis*	380	320	440	120	1.81	
	Scheloribates_ascendens	495	445	545	100	1.62	
	Eupelops_hirtus	975	850	1100	250	1.26	
	Eupelops_plicatus	590	500	680	180	1.52	
Desmonomata	*Camisia_horrida*	892.5	825	960	135	2.13	
	Camisia_invenusta	735	670	800	130	1.96	
	Camisia_segnis	865	830	900	70	2.18	
	Heminothrus_capillatus	1010	960	1060	100	1.93	*Platynothrus capillatus*
	Heminothrus_paolianus	660	640	680	40	2	*Heminothrus paolianus longisetosus*
	Platynothrus_peltifer	875	770	980	210	1.74	
	Nothrus_silvestris	760	710	810	100	1.94	
	Hermannia_gibba	860	780	940	160	1.57	
	Masthermannia_sp	442.5	400	485	85	2.47	*Masthermannia mammillaris*
	Nanhermannia_coronata	525	480	570	90	2.48	
	Nanhermannia_nana	587.5	550	625	75	2.5	
	Malaconothrus_gracilis	415	375	455	80	2.24	*Malaconothrus monodactylus*
	Trimalaconothrus_sp	525	476	574	98	2.05	mean: *T. glaber, T. angulatus, T. maior, T. foveolatus, T tardus, T. sculptus, T. vietsi*
	Mucronothrus_nasalis	690	580	800	220	2.08	
	Trhypochthoniellus_crassus	507.5	435	580	145	1.83	mean: *T. longiseta, T. setosa*
	Trhypochthonius_cladonicola	540	530	550	20	1.88	
	Trhypochthonius_tectorum	677.5	580	775	195	1.82	
Mixonomata	*Collohmannia_gigantes*	1535	1450	1620	170	1.9	
	Epilohmannia_sp	512.5	450	575	125	2.5	
	Eulomannia_ribagai	665	600	730	130	3.07	
	Rhysotritia_duplicata	580	560	600	40	1.28	
	Perlohmannia_sp	702.5	625	780	155	2.63	*Perlohmannia nasuta*
	Phthiracarus_sp	400	330	470	140	1.45	*Phthiracarus anonymys* (Grandjean 1934)[27]
	Atropacarus_striculus	465	340	590	250	1.62	Kamill & Baker 1980[28]
	Steganacarus_applicatus	750	740	760	20	1.42	Bernini & Avanzati 1989[29]
	Steganacarus_magnus	950	700	1200	500	1.58	*Steganacarus magna forma magnus* (Bernini & Avanzati 1988)[30]

Supplementary Table 4 continued

**Enarthronota**	*Atopochthonius_artiodactylus*	200	200			1.59	
	Pterochthonius_angelus	400	350	450	100	1.51	
	Brachychthonius_bimaculatus	165	157	173	16	2.5	
	Eobrachychthonius_sexnotatus	302.5	270	335	65	1.53	*Eobrachychthonius latior*
	Liochthonius_neglectus	193.5	182	205	23	2.03	
	Liochthonius_peduncularius	205	200	210	10	1.49	
	Neoliochthonius_piluliferus	194.5	123	266	143	2.18	
	Sellnickochthonius_cricoides	150	135	165	30	2.03	
	Sellnickochthonius_immaculatus	182.5	170	195	25	2.03	
	Sellnickochthonius_zelawaiensis	159	148	170	22	2.03	
	Cosmochthonius_lanatus	175	170	180	10	1.78	
	Sphaerochthonius_sp	292.5	275	310	35	1.46	*Sphaerochchthonius splendidus*
	Eniochthonius_minutissimus	375	370	380	10	2.03	
	Hypochthonius_luteus	615	580	650	70	1.97	
	Hypochthonius_rufulus	675	650	700	50	1.76	
	Lohmannia_banksi_E	1000	1000			2.16	*Lohmannia paradoxa*
	Mesoplophora_cubana	435	420	450	30	1.45	*Mesoplophora aficana* (Els 1966)[31]
**Parhypo-somata**	*Gehypochthonius_urticinus*	267.5	260	275	15	2.61	*Gehypochthonius rhadamanthus*
	Parhypochthonius_aphidinus	435	350	520	170	2.37	
**Palaeo-somata**	*Aphelacarus_sp*	440	440			2.74	*Aphelacarus acarinus*
	Ctenacarus_araneola	367.5	360	375	15	2.12	
	Palaeacarus_hystricinus	360	340	380	40	2.64	Schatz 2004[32]

Supplementary Table 5. Trophic traits used in this study and list of taxa for which stable isotope data were available. Data are from four 4 habitats[33-38] to cover a wide taxon sampling (in total 70 species) which covers most species from the morphological trait dataset and phylogeny (in total 103 species). Trophic levels were assigned based on ^{15}N values within communities (to account for site specific differences of the baseline values of ^{13}C and ^{15}N, which can geographically strongly differ) and categorized into four, very general functional groups. Within each trophic level, species were hierarchical assigned to their ^{15}N position within their community. For better visualisation in the graph showing the distribution of trophic traits along the phylogeny, we added the factor 28 to ^{13}C and the factor 8 to ^{15}N values to obtain only positive values, which are also listed in this table. Species are listed from lowest to highest trophic levels and include Brachypylina (n=49), Desmonomata (n=12), Mixonomata (n=6) and Enarthronota (n=3, including a single measurement for Brachychthoniidae).

**infraorder**	**species**	**^{13}C**	**^{15}N**	**^{13}C + 28**	**^{15}N + 8**	**trophic level**	**functional group**
Brachypylina	*Carabodes_labyrinthicus*	1.78	-4.34	29.78	3.66	0	**specialist**
Brachypylina	*Mycobates_parmeliae*	-0.03	-7.31	27.97	0.69	0.2	
Brachypylina	*Phauloppia_lucorum*	-8.66	-6.62	19.34	1.38	0.23	
Brachypylina	*Cymbaeremaeus_cymba*	-24.23	-5.06	3.77	2.94	0.6	
Brachypylina	*Anachipteria_sp*	3.34	-1.57	31.34	6.43	0.99	
Desmonomata	*Camisia_invenusta*	-25.31	-4.64	2.69	3.36	0.99	
Desmonomata	*Camisia_horrida*	4.46	-0.57	32.46	7.43	1	**primary decomposer**
Desmonomata	*Hermannia_gibba*	-24.4	-4.23	3.6	3.77	1	
Brachypylina	*Scutovertex_sculptus*	0.38	1.55	28.38	9.55	1	
Enarthronota	*Neoliochthonius_piluliferus*	1.75	2.98	29.75	10.98	1.13	
Brachypylina	*Euzetes_globulus*	4.87	0.92	32.87	8.92	1.18	
Brachypylina	*Carabodes_subarcticus*	6.8	1.17	34.8	9.17	1.36	
Brachypylina	*Tectocepheus_sarekensis*	-0.08	2.98	27.92	10.98	1.36	
Mixonomata	*Steganacarus_applicatus*	21.75	-2.67	49.75	5.33	1.37	
Brachypylina	*Limnozetes_foveolatus*	1.98	3.63	29.98	11.63	1.38	
Mixonomata	*Epilohmannia_sp*	3.15	3.79	31.15	11.79	1.44	
Brachypylina	*Chamobates_pusillus*	4.17	1.42	32.17	9.42	1.45	
Brachypylina	*Achipteria_coleoptrata*	1.07	3.23	29.07	11.23	1.5	
Brachypylina	*Hydrozetes_lacustris*	2.95	6.47	30.95	14.47	1.5	

Supplementary Table 5 continued

Desmonomata	*Malaconothrus_gracilis*	1.62	1.98	29.62	9.98	1.6	primary decomposer
Mixonomata	*Steganacarus_magnus*	8.06	2.47	36.06	10.47	1.64	
Desmonomata	*Camisia_segnis*	-26.37	-3.31	1.63	4.69	1.67	
Enarthronota	*Eniochthonius_minutissimus*	-2.09	-1.31	25.91	6.69	1.67	
Brachypylina	*Oromurcia_sudetica*	5.52	1.4	33.52	9.4	1.67	
Brachypylina	*Scheloribates_ascendens*	-0.18	-0.87	27.82	7.13	1.71	
Desmonomata	*Platynothrus_peltifer*	-4.46	-1.45	23.54	6.55	1.75	
Desmonomata	*Nanhermannia_nana*	2.08	1.57	30.08	9.57	1.76	
Brachypylina	*Galumna_lanceata*	1.72	4.42	29.72	12.42	1.89	
Brachypylina	*Liacarus_coracinus*	-11.19	1.09	16.81	9.09	1.98	
Desmonomata	*Nothrus_silvestris*	4.67	2.92	32.67	10.92	1.99	
Brachypylina	*Metabelba_papilles*	2.05	5.98	30.05	13.98	2	secondary decomposer
Mixonomata	*Rhysotritia_duplicata*	5.71	3.12	33.71	11.12	2	
Brachypylina	*Edwardzetes_edwardsi*	-27.56	-2.41	0.44	5.59	2.07	
Brachypylina	*Hermannobates_monstruosus*	-0.72	-0.41	27.28	7.59	2.13	
Brachypylina	*Eueremaeus_oblongus*	-12.93	-1.1	15.07	6.9	2.14	
Brachypylina	*Liebstadia_humerata*	-0.35	1.57	27.65	9.57	2.25	
Brachypylina	*Oribatula_tibialis*	-0.35	1.57	27.65	9.57	2.25	
Brachypylina	*Rostrozetes_fove_ovo*	2.61	5.66	30.61	13.66	2.25	
Brachypylina	*Ceratoppia_bipilis*	-12.25	-2.02	15.75	5.98	2.29	
Brachypylina	*Odontocepheus_elongatus*	5.75	3.49	33.75	11.49	2.29	
Brachypylina	*Caleremaeus_monilipes*	-12.6	-2.3	15.4	5.7	2.32	
Brachypylina	*Punctoribates_punctum*	2.54	5.93	30.54	13.93	2.42	
Brachypylina	*Cepheus_latus*	-23.22	-1.81	4.78	6.19	2.43	
Brachypylina	*Eupelops_plicatus*	1.83	6	29.83	14	2.5	
Desmonomata	*Heminothrus_paolianus*	-1.35	1.61	26.65	9.61	2.54	
Brachypylina	*Xenillus_tegeocranus*	0.38	1.6	28.38	9.6	2.55	
Brachypylina	*Pilogalumna_rosauraruizae*	1.95	5.14	29.95	13.14	2.56	
Brachypylina	*Protoribates_hakonensis*	-2.31	5.2	25.69	13.2	2.56	
Brachypylina	*Chamobates_subglobulus*	2.2	6.43	30.2	14.43	2.58	
Brachypylina	*Acrogalumna_longipluma*	-10.09	1.78	17.91	9.78	2.64	
Mixonomata	*Atropacarus_striculus*	4.6	2.15	32.6	10.15	2.66	
Brachypylina	*Eupelops_hirtus*	1.68	6.44	29.68	14.44	2.67	

Supplementary Table 5 continued

Brachypylina	*Chamobates_voigtsi*	-24.07	-0.76	3.93	7.24	2.71	**secondary decomposer**
Desmonomata	*Nanhermannia_coronata*	5	4.74	33	12.74	2.71	
Brachypylina	*Tectocepheus_minor*	1.62	6.62	29.62	14.62	2.75	
Mixonomata	*Phthiracarus_sp*	-20.93	-0.69	7.07	7.31	2.79	
Enarthronota	*Hypochthonius_rufulus*	1.92	4.96	29.92	12.96	2.87	
Brachypylina	*Disorrhina_ornata*	1.93	6.83	29.93	14.83	2.92	
Brachypylina	*Oppiella_nova*	2.57	7.15	30.57	15.15	2.99	
Brachypylina	*Oppiella_splendens*	3.53	6.04	31.53	14.04	2.99	
Brachypylina	*Damaeus_flagelloides*	-23.68	1.18	4.32	9.18	3	**predator/scavenger**
Brachypylina	*Tectocepheus_velatus*	1.55	1.9	29.55	9.9	3	
Brachypylina	*Trichoribates_trimaculatus*	6.85	7.39	34.85	15.39	3	
Brachypylina	*Oppiella_nasuta*	3.81	6.54	31.81	14.54	3.2	
Brachypylina	*Xenillus_discrepans*	1.29	2.95	29.29	10.95	3.23	
Desmonomata	*Trimalaconothrus_sp*	0.67	3.14	28.67	11.14	3.49	
Brachypylina	*Carabodes_coriaceus*	-21.74	1.65	6.26	9.65	3.5	
Brachypylina	*Damaeobelba_minnutissima*	2.2	7.45	30.2	15.45	3.5	
Desmonomata	*Trhypochthoniellus_crassus*	2.38	3.94	30.38	11.94	3.5	
Brachypylina	*Limnozetes_rugosus*	3.89	7.67	31.89	15.67	3.99	

Supplementary References

1. Pachl, P., Lindl, A. C., Krause, A., Scheu, S., Schaefer, I., Maraun, M. The tropics as ancient cradle of oribatid mite diversity. *Acarologia* 57, 309-322 (2017).
2. Schaefer, I., Norton, R. A., Scheu, S. & Maraun, M. Arthropod colonization of land – linking molecules and fossils in oribatid mites (Acari, Oribatida). *Mol. Phylogenet. Evol*. 57, 113-121 (2010).
3. Pepato, A. R. & Klimov, P. B. Origin and higher-level diversification of acariform mites – evidence from nuclear ribosomal genes, extensive taxon sampling, and secondary structure alignment. *BMC Evol. Biol.* 15, 178 (2015).
4. Krantz, G. W. & Proctor, D. E. in *A manual of Acarology*. eds Krantz GW and Walter DE (3rd edition, Texas Tech University Press 2009); chapter 15, pp. 430 ff.
5. Dabert, M., Witalinski, W., Kazmierski, A., Olszanowski, Z. & Dabert, J. Molecular phylogeny of acariform mites (Acari, Arachnida): strong conflict between phylogenetic signal and long-branch attraction artifacts. *Mol. Phylogenet. Evol*. 56, 222-241 (2010)
6. Klimov, P. B. & OConnor, B. Is permanent parasitism reversible? **-** critical evidence from early evolution of house dust mites. *Syst. Biol.* 62, 411-423 (2013).
7. Caruso, T. & Migliorini, M. Euclidean geometry explains why lengths allow precise body mass estimates in terrestrial invertebrates: the case of oribatid mites. *J Theor Biol*, **256**, 436-440 (2009).
8. Weigmann, G. Hornmilben (Oribatida) Die Tierwelt Deutschlands, 76. Teil. Goecke & Evers, Keltern (2006).
9. Grandjean, F. Essai de classification des Oribates (Acariens). *Bull. Soc. Zool. Fr.* **78**, 421-446 (1954).
10. Grandjean F. Considération sur le classement des Oribates leur division en 6 groupes majeurs. *Acarologia* **11,** 127-153 (1969).
11. Schatz, H. Die Oribatidenliteratur und die beschriebenen Oribatidenarten (1758-2001): Eine Analyse. *Abh. Ber. Naturk. Görlitz* **74,** 37-45 (2002).
12. Krivolutsky, D. A. & Krassilov, V. A. *Oribatid mites from Upper Jurassic, USSR.* In: Skarlato OA, Balasho S (Eds.), Morphology and diagnostics of mites. *Academy of Sciences of the USSR, Leningrad*, pp. 16-24 (1977).
13. Arillo, A., Subías, L. S. & Shtanchaeva, U. A new fossil oribatid mite, *Ommatocepheus nortoni* sp. nov. (Acariformes, Oribatida, Cepheidae) from a new outcrop of lower Cretaceous Àlava amber (northern Spain). *System. Appl. Acarol.* **13**, 252-255 (2008).
14. Sivhead, U. & Wallwork, J. A. An early Jurassic oribatid mite from southern Sweden. *Geol. Foren. Stock. For.* **100**, 65-70 (1978).
15. Bulanova-Zachvatkina, Y. M. A new genus of mite (Acariformes, Oribatei) from the upper Cretaecous of Taymyr. *Paleont. Journ.* **8**, 247-250 (1974).

16. Arillo, A., Subías, L. S. & Shtanchaeva, U. A new species of fossil oribatid mite (Acariformes, Oribatida, Trhypochthoniida) from the lower Cretaceous amber of San Just (Teruel Province, Spain). *System. Appl. Acarol.* **17**, 106-112 (2012).
17. Subías, L. S. & Arillo, A. Oribatid fossil mites from the upper Devonian of South Mountain, New York and the lower Carboniferous of County Antrim, North Ireland (Acariformes, Oribatida). *Estud. Museo Cienc. Nat. Àlava* **17**, 93-106 (2002).
18. Norton, R. A., Bonamo, P. M., Crierson, J. D. & Shear, W. A. Oribatid mite fossils from a terrestrial deposit near Gilboa, New York. *J. Paleontol.* **62**, 259-269 (1988).
19. Shear, W. A., Bonamo, P. M., Grierson, J. D., Rolfe, W. D. I., Laidlaw Smith, E. & Norton, R. A. Early land animals in North America: evidence from Devonian age arthropods from Gilboa, New York. *Science* **224**, 492-494 (1984).
20. O'Dowd, D. J., Brew, C. R., Christophel, D. C. & Norton, R. A. Mite-plant associations from the Eocene of Southern Australia. *Science* **252**, 99-101 (1991).
21. Woolley, T. A. Fossil oribatid mites in amber from Chiapas, Mexico (Acarina: Oribatei: Cryptostigmata). *Univ. Calif. Publ. Entomol.* **63**, 91-99 (1971).
22. Mico, L. Oribatid mite fossils from pre-Quarternary sediments in Slovenian caves III. Two new species of *Dissorhina* (Oppiidae) from the Pliocene. *Acarologia* **55**, 449-457 (2015).
23. Norton, R. A. First record of *Collohmannia* (*C. schusteri* n. sp.) and *Hermannia* (*H. sellnicki* n. sp.) from Baltic amber, with notes on Sellnick's genera of fossil oribatid mites (Acari: Oribatida). *Acarologia* **46**, 111-125 (2006).
24. Labandeira, C. C., Phillips, T. L. & Norton, R. A. Oribatid mites and the decomposition of plant tissues in Paleozoic coal-swamp forests. *Palaios* **12**, 319-353 (1997).
25. Doğan, S. & Ayyildiz, N. Systematic investigation on *Tectocepheus* Berlese, 1895 (Acari: Oribatida) species in Erzincan and Erzurum plains. *Türk. entomol. Der*. **24,** 69-80 (2000).
26. Knülle, W. Die Arten der Gattung *Tectocepheus* Berlese (Acarina: Oribatei). *Zool. Anz.* **152,** 280-305 (1953).
27. Grandjean, F. *Phthiracarus anonymum*, n. sp. *Rev. Fr. Entomol.* **1,** 51-59 (1934).
28. Kamill, B. W. & Baker, A. The genus *Atropacarus* Ewing (Acari: Cryptostigmata). *Bull. Br. Mus. Nat. Hist. (Zool.)* **39,** 189-204 (1980).
29. Bernini, F. & Avanzati, A.M. Notulae oribatologicae xlix. taxonomic revision of *Steganacarus (Steganacarus) apllicatus* (Sellnick, 1920) and the description of a new west Mediterranean Steganacarus species (Acarida, Oribatida). *Int. J. Acarol.* **15**, 65-74 (1989).
30. Bernini, F. & Avanzati, A. M. Taxonomic revision of *Steganacarus (Steganacarus) magnus* (Nicolet, 1855) (Acarida, Oribatida). *J. Nat. Hist.* **22**, 435-464 (1988).
31. Els, A. J. Studies on the South African Oribatei Inferiores (Acarina) part 1: description of *Mesoplophora africana* (Balogh 1958). *S. Afr. J. Sci.* **62,** 76-79 (1966).

32. Schatz, H. *Palaeacarus hystricinus* Trägårdh, 1932 (Acari: Oribatida: Palaeacaridae), a remarkable oribatid mite from Tyrol. *Ber. nat.-med. Verein Innsbruck*, **91,** 339-340 (2004).
33. Erdmann, G., Otte, V., Langel, R., Scheu, S. & Maraun, M. The trophic structure of bark-living oribatid mite communities analysed with stable isotopes (15N, 13C) indicates strong niche differentiation. *Exp. Appl. Acarol.* **41**, 1-10 (2007).
34. Lehmitz, R. & Maraun M. Small-scale spatial heterogeneity of stable isotopes signatures (δ15N, δ13C) in *Sphagnum* sp. Transfers to all trophic levels in oribatid mites. *Soil Biol. Biochem.* **100**, 242-251 (2016).
35. Maaß, S., Maraun, M., Scheu, D., Rillig, M. C. & Caruso, T. Environmental filtering vs. resource-based niche partitioning in diverse soil animal assemblages. *Soil Biol. Biochem.* **85**, 145-152 (2015).
36. Maraun, M., Erdmann, G., Fischer, B. M., Pollierer, M. M., Norton, R. A., Schneider, K. & Scheu, S. Stable isotopes revisited: Their use and limits for oribatid mite trophic ecology. *Soil Biol. Biochem.* **43**, 87-882 (2011).
37. Ilardi, M., Dick, J. T. A., Maraun, M. & Caruso, T. Response of native forest floor microarthropods to the invasive macroinvertebrate *Arcitalitrus dorrieni* (Crustacea; Amphipoda) (in preparation, 2019).
38. Magilton, M., Maraun, M., Emmerson, M. & Caruso, T. Oribatid mites reveal that competition for resources and trophic structure combine to regulate the assembly of diverse soil animal communities. (in preparation, 2019).
39. Original stable isotope data were obtained from the authors on request.
