# Supplementary material for: Oribatid mites show that soil food web complexity and close aboveground-belowground linkages emerged in the early Paleozoic
Source: Commun Biol. 2019 Oct 22;2:387. doi: 10.1038/s42003-019-0628-7 (PMC6805910; doi:10.1038/s42003-019-0628-7)
Supplement: Supplementary file 2 — Reporting Summary [file 42003_2019_628_MOESM2_ESM.pdf]

nature research

Corresponding author(s): Ina Schaefer

Last updated by author(s): 2019-18-08

Reporting Summary

Nature Research wishes to improve the reproducibility of the work that we publish. This form provides structure for consistency and transparency in reporting. For further information on Nature Research policies, see Authors & Referees and the Editorial Policy Checklist.

Statistics

For all statistical analyses, confirm that the following items are present in the figure legend, table legend, main text, or Methods section.

n/a	Confirmed	
☐	☒	The exact sample size (*n*) for each experimental group/condition, given as a discrete number and unit of measurement
☐	☒	A statement on whether measurements were taken from distinct samples or whether the same sample was measured repeatedly
☐	☒	The statistical test(s) used AND whether they are one- or two-sided<br>*Only common tests should be described solely by name; describe more complex techniques in the Methods section.*
☒	☐	A description of all covariates tested
☐	☒	A description of any assumptions or corrections, such as tests of normality and adjustment for multiple comparisons
☐	☒	A full description of the statistical parameters including central tendency (e.g. means) or other basic estimates (e.g. regression coefficient) AND variation (e.g. standard deviation) or associated estimates of uncertainty (e.g. confidence intervals)
☐	☒	For null hypothesis testing, the test statistic (e.g. *F*, *t*, *r*) with confidence intervals, effect sizes, degrees of freedom and *P* value noted<br>*Give P values as exact values whenever suitable.*
☐	☒	For Bayesian analysis, information on the choice of priors and Markov chain Monte Carlo settings
☒	☐	For hierarchical and complex designs, identification of the appropriate level for tests and full reporting of outcomes
☒	☐	Estimates of effect sizes (e.g. Cohen's *d*, Pearson's *r*), indicating how they were calculated

Our web collection on statistics for biologists contains articles on many of the points above.

Software and code

Policy information about availability of computer code

Data collection	no software was used for data collection
Data analysis	for data analysis open source programs were used: MrBayes v3.2.6, BEAST v2.4.7, R v3.2.4<br>all relevant parameters and commands are explained in the Material and Methods section

For manuscripts utilizing custom algorithms or software that are central to the research but not yet described in published literature, software must be made available to editors/reviewers. We strongly encourage code deposition in a community repository (e.g. GitHub). See the Nature Research guidelines for submitting code & software for further information.

Data

Policy information about availability of data

All manuscripts must include a data availability statement. This statement should provide the following information, where applicable:

- Accession codes, unique identifiers, or web links for publicly available datasets
- A list of figures that have associated raw data
- A description of any restrictions on data availability

All datasets generated or analyszed during this study are included in this published article (and its Supplementary Information files).

Field-specific reporting

Please select the one below that is the best fit for your research. If you are not sure, read the appropriate sections before making your selection.

☐ Life sciences ☐ Behavioural & social sciences ☒ Ecological, evolutionary & environmental sciences

Ecological, evolutionary & environmental sciences study design

All studies must disclose on these points even when the disclosure is negative.

Study description	Inferring the evolution of early soil food webs by trait assignment to phylogeny using published data, quantification of phylogenetic signal of traits using randomization processes and timing of trait divergence using a calibrated molecular clock
Research sample	Forphylogeny data were downloaded from NCBI, trait data were obtained from literature and by contacting aauthors that published trophic signatures (stable isotope data) of the focal taxa. The goal was to obtain as many data points for traits (global) as possible and is not restricted to any geographic context but covers, to our knowledge, the most complete phylogeny of the focal taxa currently available.
Sampling strategy	Sample size depended on alla vailable sequences on NCBI for phylogeny and of available trait data for the respective speceis/taxa represented in the phylogeny.
Data collection	Data were downloaded from NCBI, literature and on request from authors of published data as descfribed in methods.
Timing and spatial scale	Data collection from NCBI started in december 2016 and ended in march 2017, trait data collection strated in december 2016 and ended in march 2017, repeated data collection was not necessary because the most complete datasets were obtained by that point of time.
Data exclusions	DNA sequences that clustered incorrecctly in the phylogenetic tree (potential misidentifications or bad quality sequences) were excluded. Fossil calibration priors that weakened the molecular clock analysis were excluded (see Methods).
Reproducibility	All results are reproducible when following information and data provided in Material and Methods and Supplementary Information.
Randomization	Phylogenetic signal analyses we randomized trait assignment, and significance was assessed by p-values, see Results and Methods
Blinding	Blinding was not applicable for this study because trait-species assignment was specific.

Did the study involve field work? ☐ Yes ☒ No

Reporting for specific materials, systems and methods

We require information from authors about some types of materials, experimental systems and methods used in many studies. Here, indicate whether each material, system or method listed is relevant to your study. If you are not sure if a list item applies to your research, read the appropriate section before selecting a response.

Materials & experimental systems

n/a	Involved in the study
☒	☐ Antibodies
☒	☐ Eukaryotic cell lines
☒	☐ Palaeontology
☒	☐ Animals and other organisms
☒	☐ Human research participants
☒	☐ Clinical data

Methods

n/a	Involved in the study
☒	☐ ChIP-seq
☒	☐ Flow cytometry
☒	☐ MRI-based neuroimaging
